# Micronutrients in critically ill patients with severe acute kidney injury – a prospective study

**DOI:** 10.1038/s41598-020-58115-2

**Published:** 2020-01-30

**Authors:** Marlies Ostermann, Jennifer Summers, Katie Lei, David Card, Dominic J. Harrington, Roy Sherwood, Charles Turner, Neil Dalton, Janet Peacock, Danielle E. Bear

**Affiliations:** 1 King’s College London, Guy’s & St Thomas’ Foundation Hospital, Department of Critical Care, London, UK; 2 NIHR Biomedical Research Centre, Guy’s & St Thomas’ NHS Foundation Trust and King’s College London, School of Population Health and Environmental Sciences, London, UK; 3 Guy’s & St Thomas’ Foundation Hospital, Department of Critical Care, London, UK; 4 Nutristasis Unit, Viapath, St Thomas’ Hospital, London, UK; 5 King’s College Hospital NHS Foundation Trust, Department of Clinical Biochemistry, London, UK; 6 King’s College London, WellChild Laboratory, London, UK

**Keywords:** Medical research, Continuous renal replacement therapy

## Abstract

Malnutrition is common in patients with acute kidney injury (AKI) and the risk of mortality is high, especially if renal replacement therapy is needed. Between April 2013 through April 2014, we recruited critically ill adult patients (≥18 years) with severe AKI in two University hospitals in London, UK, and measured serial plasma concentrations of vitamin B_1_, B_6_, B_12_, C and D, folate, selenium, zinc, copper, iron, carnitine and 22 amino acids for six consecutive days. In patients receiving continuous renal replacement therapy (CRRT), the concentrations of the same nutrients in the effluent were also determined. CRRT patients (n = 31) had lower plasma concentrations of citrulline, glutamic acid and carnitine at 24 hrs after enrolment and significantly lower plasma glutamic acid concentrations (74.4 versus 98.2 μmol/L) at day 6 compared to non-CRRT patients (n = 24). All amino acids, trace elements, vitamin C and folate were detectable in effluent fluid. In >30% of CRRT and non-CRRT patients, the plasma nutrient concentrations of zinc, iron, selenium, vitamin D_3_, vitamin C, trytophan, taurine, histidine and hydroxyproline were below the reference range throughout the 6-day period. In conclusion, altered micronutrient status is common in patients with severe AKI regardless of treatment with CRRT.

## Introduction

Critically ill patients with acute kidney injury (AKI) are at risk of malnutrition due to several factors, including pre-existing malnourishment, chronic comorbidities, poor nutritional intake, uraemia and the effects of critical illness in general^1–4^. In patients treated with renal replacement therapy (RRT), unrecognised losses of nutrients may also contribute^5–13^. The exact degree of nutrient loss during modern RRT is unknown since most data stem from a few small studies with relatively short periods of RRT (i.e. 24 hours or less)^6–12,14–17^. In 1999, Story *et al*. found that eight patients treated with continuous renal replacement therapy (CRRT) had lower serum concentrations of selenium (Se), zinc (Zn), vitamin C and vitamin E than healthy volunteers^7^. In 2004, Berger *et al*. demonstrated significant concentrations of Cu, Se, Zn and vitamin B_1_ in effluent fluid after 8 hours of CRRT^8^. Amino acid losses up to 10–15 g/d during RRT have been reported^14^. A recent study showed that 80% of patients on CRRT had below-normal levels of at least one micronutrient i.e. thiamine, pyridoxine, ascorbic acid, folate, zinc or copper^13^.

It is unclear whether these alterations of nutrient status contribute to the poor outcomes of patients with AKI. Furthermore, it is not clear whether there is a role for routine micronutrient supplementation^18^. Guidance from official authorities and experts varies and clinical practice is variable^16,19–21^. More data, including prospective studies and intervention trials are urgently needed^13,18,19^. In preparation, more information about the micronutrient status of AKI patients receiving modern critical care is required.

We hypothesized that patients treated with CRRT had significantly lower plasma concentrations of water soluble nutrients than AKI patients without CRRT. Our objectives were:

(i) To perform serial measurements of plasma concentrations of amino acids, trace elements and vitamins for up to 6 days in critically ill patients with severe AKI and to compare CRRT patients with non-CRRT patients;
(ii) To investigate the types of nutrients that are removed during CRRT and to quantify the losses; and
(iii) To determine the proportion of patients with plasma nutrient concentrations below the reference range.

## Results

**Figure 1:**
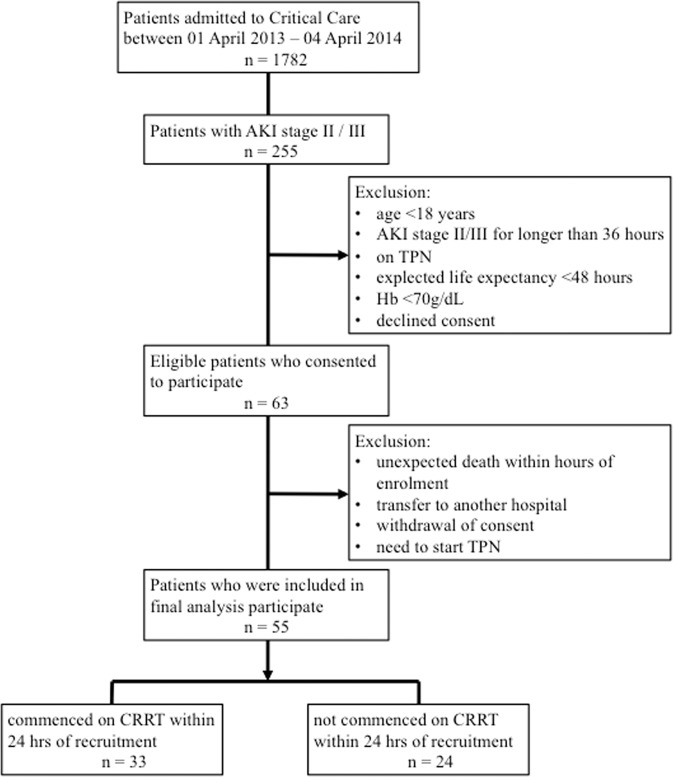
Flow chart. Abbreviations AKI = acute kidney injury; CRRT = continuous renal replacement therapy; Hb = hemoglobin; TPN = total parenteral nutrition.

Between April 1, 2013, through April 1, 2014, 63 consecutive patients were recruited (Fig. 1). Following exclusion of eight patients [unexpected death within hours of enrolment (n = 3), transfer to another hospital (n = 1), withdrawal of consent (n = 1), and need to start TPN (n = 3)], 55 patients were analysed (33 patients in the CRRT group and 24 in the non-CRRT group). The main indications for CRRT were management or prevention of pulmonary oedema (57%) and severe metabolic acidosis (47%). During the 6-day follow-up period, 12 non-CRRT patients were commenced on CRRT, and 18 CRRT patients discontinued CRRT. As such, 12 patients had CRRT every day, 12 patients did not have CRRT on any day, and 19 patients had CRRT on some but not all 6 days.

### Patient demographics

There were no clinically important differences in baseline characteristics between the CRRT and non-CRRT group on admission to the ICU (Table 1). In AKI patients receiving CRRT at enrolment, major surgery was the most common reason for admission (45%) compared to sepsis in patients with AKI not receiving CRRT (38%). ICU, hospital and 90-day mortality and length of stay were not significantly different between both groups (Table 1). There was no significant difference in protein and caloric intake between the CRRT and non-CRRT cohort during the first 24-hour period after enrolment (Table 2). The SOFA score was higher in those treated with CRRT but CRP and WBC were not significantly different.

**Table 1:** Baseline demographics.

Parameter	CRRT * (n = 31)	Non-CRRT * (n = 24)	Difference
Age [years], mean (SD)	62.3 (16.5)	58.3 (15.1)	95% CI: −4.71 to 12.64
Female gender, n (%)	10 (32%)	11 (45%)	95% CI: −0.40 to 0.12
Weight [kg], mean (SD)	83.4 (19.8)	87.4 (24.4)	95% CI: −16.58 to 8.56
Height [cm], mean (SD)	169.2 (10.1)	171.3 (10.5)	95% CI: −8.10 to 3.74
BMI, mean (SD)	29.2 (6.3)	29.7 (8.6)	95% CI: −4.75 to 3.72
Ethnicity, n (%)			
Caucasian	23 (74%)	12 (50%)	Fisher’s exact p = 0.09
Afro-Caribbean	3 (9.7%)	1 (4.2%)	
Asian	1 (3.2%)	5 (21%)	
Other	4 (13%)	6 (19%)	
**Factors on admission to ICU**			
Admission diagnosis, n (%)			
Post-major surgery	14 (45%)	5 (21%)	Fisher’s exact p = 0.02
Sepsis	7 (23%)	9 (38%)	
Respiratory failure	2 (6.5%)	4 (25%)	
Vasculitis	1 (3.2%)	1 (4.2%)	
Multi-organ failure	4 (13%)	0	
Neurological emergency	1 (3.2%)	1 (4.2%)	
Cardiac arrest	1 (3.2%)	0	
Other	1 (3.2%)	4 (17%)	
APACHE II score, mean (SD)	20.4 (5.3)	18.3 (5.1)	95% CI: −0.69 to 4.97
SOFA score, mean (SD)	8.3 (2.6)	7.6 (2.1)	95% CI: −0.78 to 2.34
**Parameters at enrolment**			
Serum creatinine [µmol/L], mean (SD)	266.9 (104.7)	249.1 (103.9)	p = 0.53
Serum potassium [mmol/L], mean (SD)	5.1 (0.7)	4.6 (0.6)	p = 0.009
Arterial pH, mean (SD)	7.33 (0.08)	7.37 (0.06)	p = 0.13
ARDS	27.3%	41%	p = 0.58
Mechanical ventilation	60.6%	66.7%	p = 0.78
Septic shock	30.3%	33%	p = 0.99
Treatment with catecholamines and/or vasopressor	69.7%	50%	p = 0.18
**Outcome**			
Mortality			
ICU mortality	26%	4.2%	95% CI: 0.04 to 0.39
Hospital mortality	35%	17%	95% CI: −0.05 to 0.41
90 day mortality	35%	21%	95% CI: −0.88 to 0.38
Length of stay [days]			
in ICU, mean (SD)	18.3 (16.0)	15.6 (16.7)	p = 0.40
in hospital, mean (SD)	55.6 (41.8)	45.1 (41.5)	p = 0.12

Abbreviations: APACHE = Acute Physiology and Chronic Health Evaluation; ARDS = acute respiratory distress syndrome; BMI = body mass index; CI = confidence interval; CRRT = continuous renal replacement therapy; ICU = intensive care unit; SOFA = Sequential Organ Failure Assessment; SD = standard deviation.

*As per clinical team on day of enrolment.

**Table 2:** Difference in essential vitamins, trace elements and amino acids at 24 hours after enrolment using planned treatment (intention to treat analysis).

Parameter	CRRT* (n = 31)	Non-CRRT* (n = 24)	Difference (95% CI for parametric)
Caloric intake (kcal)	836 (IQR 1523)	1300 (IQR1588)	p = 0.83
Protein intake (g)	32.1 (IQR 77.3)	63.0 (IQR 67.8)	p = 0.60
SOFA score	10.6 (SD 3.5)	8.0 (SD 3.1)	**95% CI: 0.8 to 4.4†**
**Amino acids**^**‡**^			
Leucine	120.3 (SD 60.3)	123.5 (SD 48.0)	95% CI: −33.6 to 27.1^†^
Valine	173.3 (SD 77.0)	205.7 (SD 73.0)	95% CI: −73.7 to 9.0^†^
Isoleucine	59.6 (IQR 48.0)	61.0 (IQR 32.0)	p = 0.85
Glycine	156.5 (IQR 78.0)	155.7 (IQR 95.0)	p = 0.83
Alanine	239.7 (IQR 177.0)	241.4 (IQR 105.0)	p = 0.70
Methionine	27.3 (IQR 15.0)	21.7 (IQR (13.0)	p = 0.94
Arginine	43.2 (IQR 20.0)	48.6 (IQR 21.0)	p = 0.49
Citrulline	20.4 (IQR 12.0)	20.8 (IQR 21.0)	p = 0.21
Ornithine	60.5 (SD 21.5)	64.1 (SD 28.0)	95% CI: −18.8 to 8.8^†^
Proline	120.8 (IQR 34.0)	162.7 (IQR 132.0)	p = 0.11
Glutamic acid	53.5 (IQR 34.0)	76.7 (IQR 35.0)	**p** = **0.04**
Glutamine	385.3 (SD 139.0)	405.2 (SD 148.8)	95% CI: −98.8 to 58.9^†^
Lysine	135.2 (IQR 66.0)	146.0 (IQR 72.0)	p = 0.73
Tryptophan	23.4 (IQR 15.0)	23.1 (IQR 17.0)	p = 0.68
Phenylalanine	81.8 (IQR 42.0)	89.4 (IQR 37.0)	p = 0.30
Tyrosine	63.9 (SD 27.8)	67.0 (SD 31.9)	95% CI: −19.3 to 13.2^†^
Hydroxyproline	6.4 (IQR 8.0)	7.0 (IQR 9.0)	p = 0.85
Threonine	75.1 (IQR 44.0)	68.4 (IQR 44.0)	p = 0.89
Serine	71.0 (SD 30.2)	73.1 (SD 26.6)	95% CI: −17.9 to 13.6^†^
Aspartic acid	7.1 (IQR 4.0)	7.1 (IQR 5.0)	p = 0.60
Taurine	30.9 (IQR 20.0)	30.1 (IQR 27.0)	p = 0.86
Histidine	62.6 (25.0)	57.6 (IQR 26.0)	p = 0.20
Carnitine	49.7 (IQR 20.0)	67.1 (IQR 65.0)	**p** = **0.01**
**Trace elements**^**‡**^			
Fe	6.0 (IQR 10.0)	4.6 (IQR 9.0)	p = 0.85
Se	0.6 (IQR 0.0)	0.7 (IQR 0.0)	p = 0.18
Cu	16.7 (SD 5.4)	17.2 (SD 4.0)	95% CI: −3.2 to 2.2^†^
Zn	5.4 (IQR 3.0)	5.5 (IQR 4.0)	p = 0.46
**Vitamins**			
Vitamin D_3_ [nmol/L]	15.3 (IQR 15.0)	21.5 (IQR 13.0)	p = 0.14
Folate [μg/L]	3.5 (IQR 5.0)	4.8 (IQR 5.0)	p = 0.30
Vitamin B_12_ [ng/L]	738.0 (IQR 1286.0)	757.0 (IQR 1597)	p = 0.73
Vitamin B_1_ [nmol/L]	139.7 (IQR 92.6)	121.4 (IQR 49.5)	p = 0.49
Vitamin B_6_ [nmol/L]	93.7 (IQR 108.5)	78.7 (IQR 59.6)	p = 0.24
Vitamin C [μmol/L]	9.2 (IQR 13)	16.8 (IQR 13.0)	p = 0.09

Abbreviations: CI = confidence interval; CRRT = continuous renal replacement therapy; IQR = interquartile range; SOFA = Sequential Organ Failure Assessment; Fe = iron; Se = selenium; Cu = copper; Zn = Zinc.

All non-parametric tests presented with medians (IQR), and all parametric tests (denoted with^†^) presented with mean (SD). All parametric tests have 95% confidence intervals provided.

*As per clinical team on day of enrolment.

^†^Parametric test.

^‡^In μmol/L.

### Nutrient concentrations at 24 hours after enrolment

Twenty-four hours after enrolment, CRRT patients had significantly lower plasma concentrations of glutamic acid and carnitine than non-CRRT patients. There were no other statistically significant differences (Table 2).

### Nutrient concentrations at day 6 after enrolment

Data were available for 43 patients. Intention-to-treat analysis confirmed significantly lower serum glutamic acid concentrations on day 6 in CRRT patients compared to non-CRRT patients (74.4 versus 98.2 μmol/L, respectively) (Table 3). Although the mean values of most other amino acids, trace elements and vitamins were lower in CRRT patients, the differences were statistically not significant. Analysis according to actual treatment (ie CRRT on all 6 days, CRRT on some days or no CRRT during 6 day period) received revealed no significant differences in nutrient concentrations between the three groups (Table 4).

**Table 3:** Difference in essential vitamins, trace elements and amino acids at six days after enrolment by intention to treat.

Parameter	CRRT* (n = 25)	Non-CRRT* (n = 18)	Difference (95% CI for parametric)
Caloric intake (kcal)^†^	1540 (SD 702)	1931 (SD 284)	95% CI: −794 to 12.8
Protein intake (g)^†^	63.6 (SD 31.4)	77.7 (SD 14.9)	95% CI: −33 to 4.2
SOFA score	8.0 (IQR 4.0)	6.0 (IQR 2.5)	p = 0.15
**Amino acids**^**‡**^			
Leucine	112.5 (SD 28.1)	124.7 (SD 50.5)	95% CI: −36.7 to 12.4^†^
Valine	197.1 (SD 48.9)	211.2 (SD 76.9)	95% CI: −53.3 to 25.1^†^
Isoleucine	61.8 (SD 14.1)	65.6 (SD 25.8)	95% CI: −16.3 to 8.7^†^
Glycine	183.0 (SD 75.3)	189.7 (SD 71.2)	95% CI: −53.5 to 40.1^†^
Alanine	236.1 (SD 89.1)	255.4 (SD 49.8)	95% CI: −67.5 to 28.9^†^
Methionine	26.3 (SD 13.4)	26.5 (SD 8.2)	95% CI: −7.5 to 7.3^†^
Arginine	54.0 (SD 20.1)	65.2 (SD 19.7)	95% CI: −23.9 to 1.5^†^
Citrulline	22.2 (SD 8.0)	26.2 (SD 10.0)	95% CI: −9.6 to 1.7^†^
Ornithine	73.6 (SD 31.1)	73.1 (SD 25.6)	95% CI: −17.9 to 18.9^†^
Proline	144.0 (SD 67.2)	170.2 (SD 66.4)	95% CI: −68.7 to 16.3^†^
Glutamic acid	74.4 (SD 25.0)	98.2 (SD 44.0)	95% CI: −45.6 to −2.0^†^
Glutamine	388.8 (SD 113.1)	394.8 (SD 90.1)	95% CI: −72.4 to 60.4^†^
Lysine	155.4 (SD 60.6)	170.4 (SD 51.8)	95% CI: −51.3 to 21.5^†^
Tryptophan	31.8 (SD 9.9)	27.9 (SD 10.7)	95% CI: −2.6 to 10.4^†^
Phenylalanine	99.6 (SD 36.6)	94.3 (SD 32.5)	95% CI: −17.0 to 27.5^†^
Tyrosine	74.5 (SD 27.7)	64.1 (SD 25.2)	95% CI: −6.5 to 27.5^†^
Hydroxyproline	6.2 (IQR 8.0)	4.7 (IQR 3.8)	p = 0.82
Threonine	95.5 (SD 53.1)	103.7 (SD 31.6)	95% CI: −37.2 to 20.9^†^
Serine	74.2 (IQR 42.3)	80.8 (IQR 23.8)	p = 0.59
Aspartic acid	7.4 (IQR 5.1)	8.4 (IQR 4.3)	p = 0.252
Taurine	26.6 (IQR 19.0)	22.4 (IQR 21.6)	p = 0.742
Histidine	55.8 (IQR 25.0)	54.3 (IQR 18.3)	p = 0.919
Carnitine	56.8 (SD 29.6)	49.8 (SD 18.6)	95% CI: −9.3 to 23.4^†^
**Trace elements**^**‡**^			
Fe	8.1 (IQR 7.0)	7.3 (IQR 7.0)	p = 0.79
Se	0.7 (IQR 0.0)	0.9 (IQR 0.0)	p = 0.99
Cu	17.4 (SD 5.9)	17.7 (SD 5.0)	95% CI: −3.9 to 3.1^†^
Zn	8.8 (SD 3.3)	8.3 (SD 2.4)	95% CI: −1.4 to 2.4^†^
**Vitamins**			
Vitamin D_3_ [nmol/L]	23.0 (IQR 15.0)	19.6 (IQR 14.0)	p = 0.34
Folate [μg/L]	6.3 (IQR 5.0)	5.8 (IQR 5.0)	p = 0.54
Vitamin B_12_ [ng/L]	982.0 (IQR 0.2)	837.0 (IQR 1098.0)	p = 0.79
Vitamin B_1_ [nmol/L]	171.6 (IQR 3.0)	152.9 (IQR 86.8)	p = 0.77
Vitamin B_6_ [nmol/L]	112.6 (IQR 67.9)	98.1 (IQR 49.3)	p = 0.31
Vitamin C [μmol/L]	14.8 (IQR 13.0)	17.6 (IQR 17.0)	p = 0.33

Abbreviations: CI = confidence interval; CRRT = continuous renal replacement therapy; IQR = interquartile range; SOFA = Sequential Organ Failure Assessment; Fe = iron; Se = selenium; Cu = copper; Zn = Zinc.

All non-parametric tests presented with medians (IQR), and all parametric tests (denoted with^†^) presented with mean (SD). All parametric tests have 95% confidence intervals provided.

*As per clinical team on day of enrolment.

^†^Parametric test.

^‡^In μmol/L.

**Table 4:** Difference in essential vitamins, trace elements and amino acids at six days after enrolment using actual treatment.

Parameter	CRRT* (n = 12)	Non-CRRT* (n = 12)	Mixed* (n = 31)	Differences
Caloric intake (kcal)	1650 (IQR 1455)	1860 (IQR 485)	1863 (IQR 695)	**p** = **0.03**
	CRRT vs non-CRRT CRRT vs Mixed Non-CRRT vs Mixed			**95% CI: −1204 to −32** 95% CI: −943 to 28 95% CI: −359 to 680
Protein intake (g)	63.0 (IQR 67.2)	74.0 (IQR 13.1)	76.8 (IQR 22.1)	**p** = **0.03**
	CRRT vs non-CRRT CRRT vs Mixed Non-CRRT vs Mixed			**95% CI: −53 to −1.1** **95% CI: −43 to −0.04** 95% CI: −17 to 28
SOFA score	9.0 (IQR 8.0)	5.0 (IQR 1.5)	7.0 (IQR 3.0)	**p** = **0.04**
	CRRT vs non-CRRT CRRT vs Mixed Non-CRRT vs Mixed			95% CI: 0.3 to 7.2 95% CI: −1.1 to 4.7 95% CI: −4.8 to 0.9
**Amino acids**^**†**^				
Leucine	109.5 (IQR 53.0)	127.4 (IQR 95.0)	127.1 (IQR 60.0)	p = 0.3
Valine	173.2 (IQR 121.0)	215.8 (IQR 126.0)	214.5 (IQR 82.0)	p = 0.3
Isoleucine	62.2 (IQR 20.0)	60.5 (IQR 50.0)	62.3 (IQR 34.0)	p = 0.6
Glycine	163.1 (IQR 111.0)	179.9 (IQR 137.0)	181.0 (IQR 64.0)	p = 0.9
Alanine	224.8 (IQR 135.0)	262.1 (IQR 104.0)	262.7 (IQR 105.0)	p = 0.2
Methionine	28.2 (IQR 24.0)	25.3 (IQR 11.0)	26.4 (IQR 11.0)	p = 0.5
Arginine	47.7 (IQR 38.0)	61.7 (IQR 30.0)	50.4 (IQR 28.0)	p = 0.1
Citrulline	20.1 (IQR 4.0)	27.0 (IQR 11.0)	30.6 (IQR 15.0)	p = 0.2
Ornithine	79.5 (IQR 59.0)	76.7 (IQR 38.0)	71.5 (IQR 20.0)	p = 0.8
Proline	146.2 (IQR 106.0)	146.4 (IQR 64.0)	161.9 (IQR 63.0)	p = 0.6
Glutamic acid	57.9 (IQR 43.0)	77.8 (IQR 48.0)	85.9 (IQR 42.0)	p = 0.2
Glutamine	352.0 (IQR 139.0)	382.1 (IQR 153.0)	400.5 (IQR 131.0)	p = 0.9
Lysine	149.4 (IQR 157.0)	163.1 (IQR 60.0)	162.5 (IQR 70.0)	p = 0.7
Tryptophan	30.8 (IQR 19.0)	29.7 (IQR 14.0)	35.1 (IQR 14.0)	p = 0.3
Phenylalanine	78.1 (IQR 85.0)	87.6 (IQR 58.0)	98.9 (IQR 35.0)	p = 0.8
Tyrosine	69.6 (IQR 61.0)	57.5 (IQR 39.0)	77.2 (IQR 27.0)	p = 0.3
Hydroxyproline	3.4 (IQR 11.0)	5.8 (IQR 5.0)	11.9 (IQR 4.0)	p = 0.9
Threonine	86.1 (IQR 72.0)	101.0 (IQR 65.0)	94.0 (IQR 51.0)	p = 0.8
Serine	82.0 (IQR 48.0)	77.3 (IQR 27.0)	75.2 (IQR 17.0)	p = 0.5
Aspartic acid	8.4 (IQR 8.0)	8.7 (IQR 4.0)	7.2 (IQR 4.0)	p = 0.4
Taurine	20.0 (IQR 15.0)	21.9 (IQR 20.0)	27.3 (IQR 32.0)	p = 0.7
Histidine	55.8 (IQR 34.0)	54.6 (IQR 27.0)	56.8 (IQR 15.0)	p = 0.9
Carnitine	43.3 (IQR 58.0)	59.8 (IQR 30.0)	60.8 (IQR 42.0)	p = 0.4
**Trace elements**^**†**^				
Fe	8.1 (IQR 5.0)	7.0 (IQR 7.0)	7.6 (IQR 6.0)	p = 0.5
Se	0.7 (IQR 1.0)	0.9 (IQR 0.0)	0.8 (IQR 0.0)	p = 0.7
Cu	18.3 (IQR 9.0)	19.8 (IQR 7.0)	17.0 (IQR 7.0)	p = 0.8
Zn	9.4 (IQR 4.0)	7.4 (IQR 4.0)	8.5 (IQR 4.0)	p = 0.1
**Vitamins**				
Vitamin D_3_ [nmol/L]	21.1 (38.0)	19.9 (IQR 15.0)	19.7 (IQR 13.0)	p = 0.9
Folate [μg/L]	6.3 (IQR 10.0)	5.9 (IQR 6.0)	6.0 (IQR 5.0)	p = 0.7
Vitamin B_12_ [ng/L]	1336.0 (IQR 652.0)	843.5 (IQR 1238.0)	800.0 (IQR 881.0)	p = 0.1
Vitamin B_1_ [nmol/L]	171.6 (IQR 103)	139.0 (IQR 49.0)	133.5 (IQR 65.0)	p = 0.7
Vitamin B_6_ [nmol/L]	112.6 (IQR 82.0)	79.7 (IQR 34.0)	85.2 (IQR 49.0)	p = 0.5
Vitamin C [μmol/L]	10.1 (IQR 12.0)	19.0 (IQR 19.0)	19.3 (IQR 17.0)	p = 0.7

Abbreviations: CI = confidence interval; CRRT = continuous renal replacement therapy; IQR = interquartile range; SOFA = Sequential Organ Failure Assessment; Fe = iron; Se = selenium; Cu = copper; Zn = Zinc.

All non-parametric results presented with medians (IQR).

*As per clinical team on day of enrolment.

^†^In μmol/L.

### Clearance of nutrients during CRRT

In CRRT patients, the mean dose of delivered RRT in the first 24 hours after enrolment was 46.1 L (SD 14.8) and the mean RRT dose during the whole period of RRT was 34.5 ml/kg/hr (SD 8.4). All amino acids, trace elements, and water soluble vitamins were detectable in effluent fluid (Table 5). Vitamin B1, B6, B12 and D were not detectable.

**Table 5:** Losses of essential vitamins, trace elements and amino acids in effluent fluid at 24 hours after starting continuous renal replacement therapy.

CRRT group (n = 31)	Mean concentration (SD) in effluent at 24 hours	Total loss in first 24 hours of CRRT*	Range of total losses in first 24 hours of CRRT
**Amino acids**			
Leucine [μmol/L]	112.5 (48.3)	4299 (2650)	1353–14174 [μmol/24 h]
Valine [μmol/L]	185.8 (68.0)	7670 (5271)	2738–23207 [μmol/24 h]
Isoleucine [μmol/L]	71.4 (27.7)	2788 (1870)	848–8516 [μmol/24 h]
Glycine [μmol/L]	168.6 (58.4)	7120 (8115)	1234–18790 [μmol/24 h]
Alanine [μmol/L]	280.0 (104.8)	10779 (12137)	2802–32811 [μmol/24 h]
Methionine [μmol/L]	28.4 (11.3)	1098 (1297)	111–4705 [μmol/24 h]
Arginine [μmol/L]	59.1 (22.7)	2256 (1713)	10–7949 [μmol/24 h]
Citrulline [μmol/L]	19.0 (7.1)	834 (SD 393)^†^	141–1556 [μmol/24 h]
Ornithine [μmol/L]	55.2 (24.0)	2189 (1995)	19–7145 [μmol/24 h]
Proline [μmol/L]	109.0 (54.5)	4430 (5637)	41–11938 [μmol/24 h]
Glutamic acid [μmol/L]	33.0 (3.5)	1453 (1091)	1–3908 [μmol/24 h]
Glutamine [μmol/L]	409.8 (24.5)	17242 (17140)	203–38532 [μmol/24 h]
Lysine [μmol/L]	160.1 (17.3)	6742 (5976)	1–19099 [μmol/24 h]
Tryptophan [μmol/L]	14.7 (1.3)	587 (622)	1–1807 [μmol/24 h]
Phenylalanine [μmol/L]	92.3 (9.3)	3403 (3875)	1–13801 [μmol/24 h]
Tyrosine [μmol/L]	70.4 (6.2)	2895 (2705)	1–7553 [μmol/24 h]
Hydroxyproline [μmol/L]	40.4 (5.9)	1114 (2165)	1–9546 [μmol/24 h]
Threonine [μmol/L]	89.7 (7.6)	3420 (4289)	1–11232 [μmol/24 h]
Serine [μmol/L]	74.2 (4.0)	3300 (3252)	1–7334 [μmol/24 h]
Aspartic acid [μmol/L]	5.6 (1.3)	239 (181)	1–7334 [μmol/24 h]
Taurine [μmol/L]	23.1 (25.5)	547 (847)	1–4521 [μmol/24 h]
Histidine [μmol/L]	62.7 (27.0)	2287 (1859)	42–6332 [μmol/24 h]
Carnitine [μmol/L]	38.6 (3.9)	1540 (SD 763)^†^	38–2941 [μmol/24 h]
**Trace elements**			
Fe [μmol/L]	0.1 (0.1)	0.01 (4)	0.01–6 [μmol/24 h]
Se [μmol/L]	0.01 (0.01)	0.5 (1)	0.01–2 [μmol/24 h]
Cu [μmol/L]	0.1 (0.02)	4.9 (5)	0.01–14 [μmol/24 h]
Zn [μmol/L]	0.1 (0.2)	0.01 (11)	0.01–88 [μmol/24 h]
**Vitamins**			
Vitamin D_2_ [nmol/L]	0	0	0
Vitamin D_3_ [nmol/L]	0	0	0
Folate [μg/L]	3.1 (3.3)	96 (61)	0.01–1168 [μg/24 h]
Vitamin B_12_ [ng/L]	0	0	0
Vitamin B_1_ [nmol/L]	0	0	0
Vitamin B_6_ [nmol/L]	0	0	0
Vitamin C [μmol/L]	17.9 (18.3)	395 (870)	0.01–4172 [μmol/24 h]

Abbreviations: CRRT = continuous renal replacement therapy; SD = standard deviation; IQR = interquartile range.

*Median value and interquartile range unless indicated specifically.

^†^Mean value (standard deviation).

### Nutrient concentrations below the reference range

**Figure 2:**
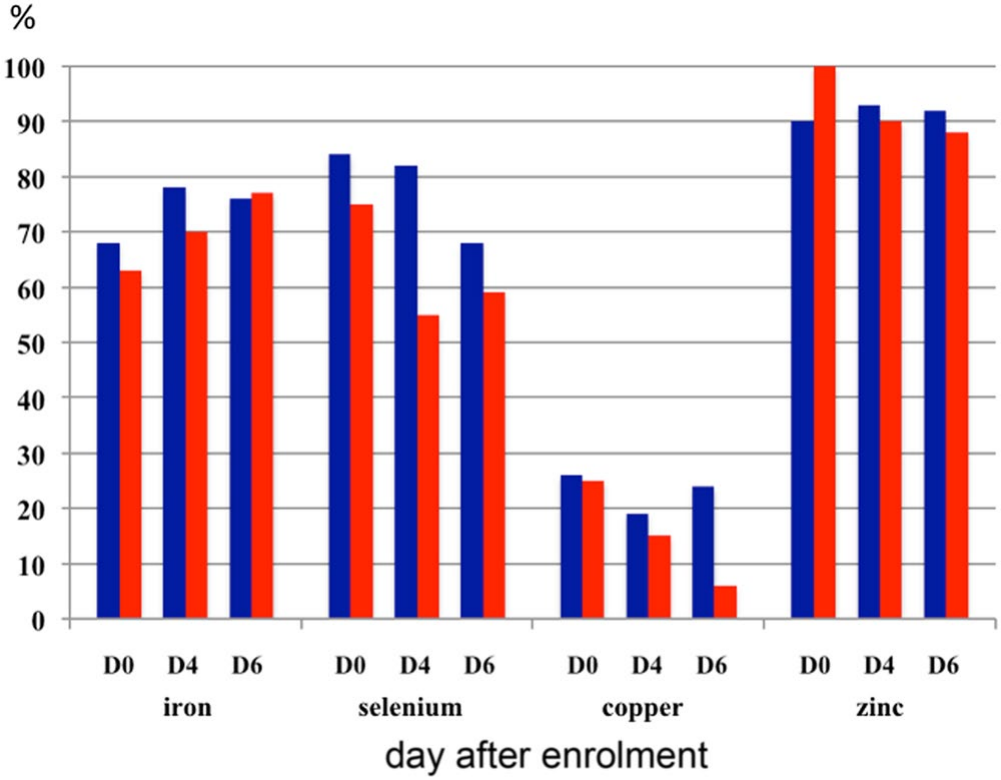
Proportion of patients with plasma concentrations of trace elements below the reference range. CRRT patients in blue; severe AKI patients who were not treated with CRRT in red.

**Figure 3:**
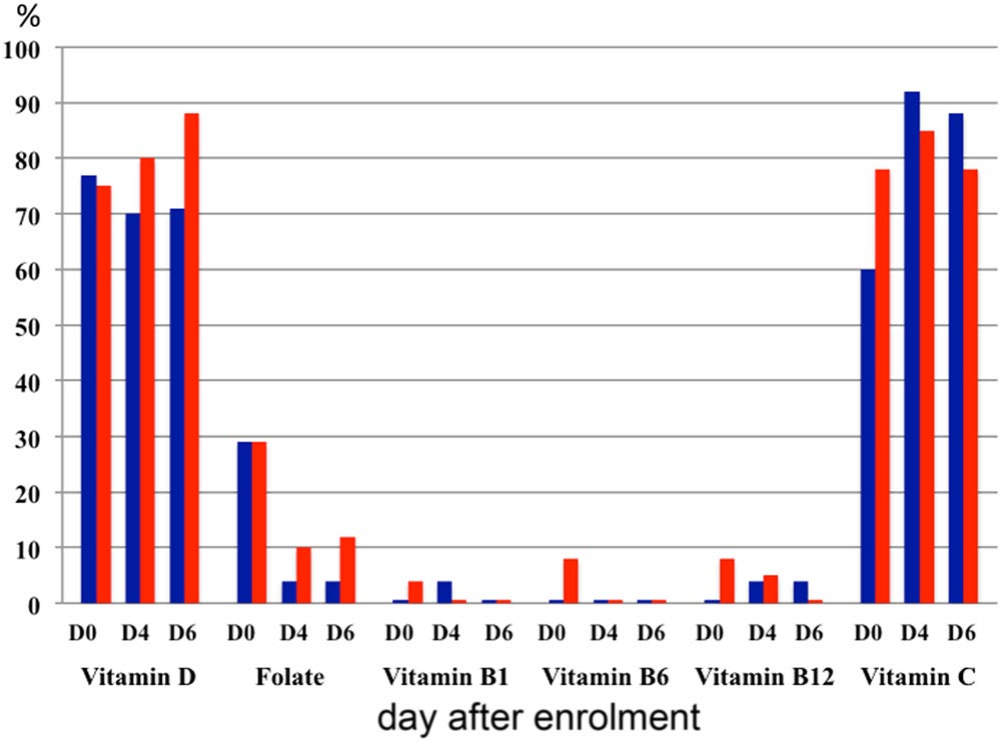
Proportion of patients with plasma concentrations of vitamins below the reference range. CRRT patients in blue; severe AKI patients who were not treated with CRRT in red.

In the majority of patients, serial plasma concentrations of vitamins and trace elements were below the reference range throughout the six-day observation period, especially Zn, Fe, Se, vitamin D3 and vitamin C, irrespective of CRRT. (Figs. 2 and 3 and Supplementary Table 2).

**Figure 4:**
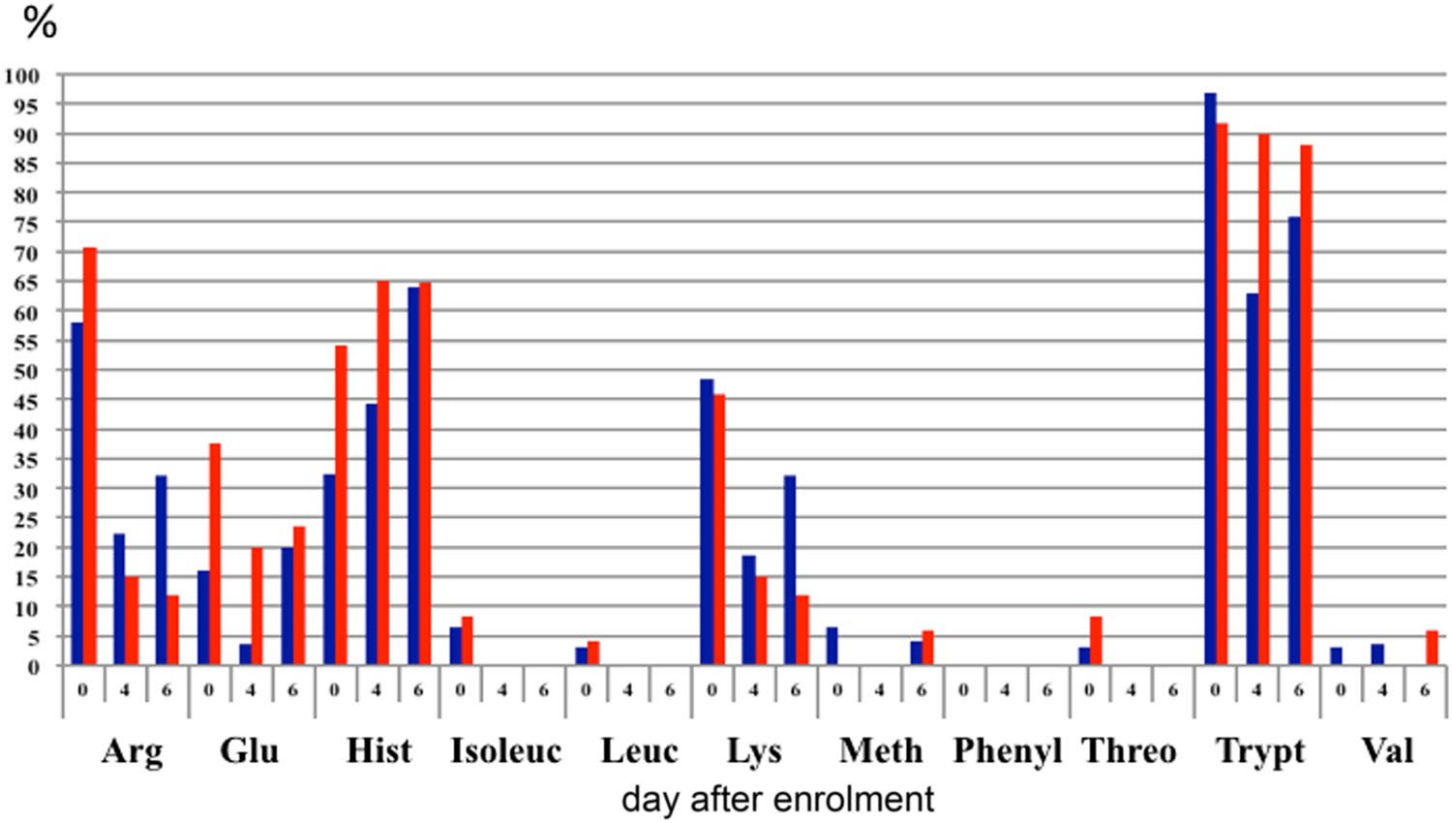
Proportion of patients with plasma concentrationas of essential and conditionally essential amino acids below the reference range. CRRT patients in blue; severe AKI patients who were not treated with CRRT in red. Abbreviations: Arg = arginine; Glu = glutamine; Hist = histidine; Isoleuc = isoleucine; Leuc = leucine; Lys = lysine; Meth = methionine; Phenyl = phenylalanine; Threo = threonine; Trypt = tryptophan; Val = valine.

**Figure 5:**
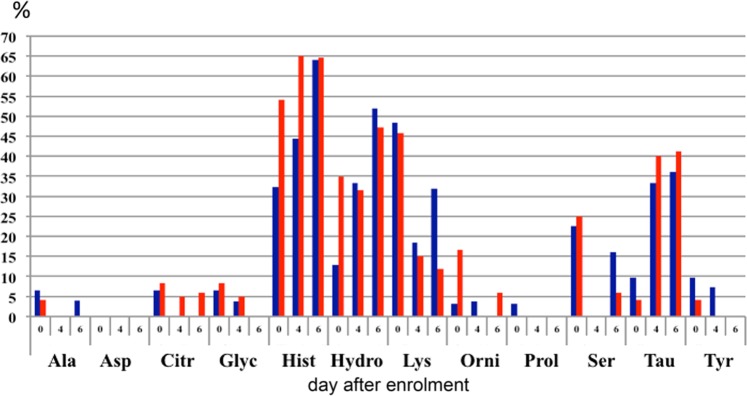
Proportion of patients with plasma concentrations of non-essential amino acids below the reference range. CRRT patients in blue; severe AKI patients who were not treated with CRRT in red. Abbreviations: Ala = alanine; Asp = aspartic acid; Citr = citrulline; Glyc = glycine; Hist = histidine; Hydro = hydroxyproline; Lys = Lysine; Orni = ornithine; Prol = proline; Ser = serine; Tau = taurine; Tyr = tyrosine.

Apart from glutamic acid and tryptophan, the mean concentrations of all amino acids were within the reference range throughout the whole six-day follow-up period. (Supplementary Table 2) However, large proportions of patients had amino acid concentrations below the reference range, most notably trytophan, taurine, serine, glutamine, citrulline, histidine, hydroxyproline, lysine and arginine (Figs. 4 and 5 and Supplementary Table 3). There was no statistically significant difference between the CRRT and non-CRRT group.

## Discussion

Our study confirms that the majority of critically ill patients with AKI had an altered micronutrient status with a large proportion of patients having nutrient concentrations below the reference range, irrespective of CRRT. With the exception of glutamic acid, there were no significant differences in plasma nutrient concentrations between the CRRT and non-CRRT group during a six-day observational period. All water soluble nutrients were detectable in the effluent in variable amounts.

It is thought that nutrient losses during RRT play an important role in the development of malnutrition in AKI^18^. Indeed, several small, observational studies in patients receiving CRRT reported losses of vitamins and micronutrients into the effluent^7,8,17^. In each of these studies, RRT modality and study duration differed and the number of measured nutrients was limited. Our study is the first where nutrient concentrations were measured over a six-day period in patients receiving modern critical care. We showed that water soluble nutrients were indeed lost into the effluent in variable quantities and that a large proportion of patients had plasma nutrient concentrations below the reference range. However, there was no statistically significant difference between the CRRT and non-CRRT group. In addition, low nutrient concentrations were seen at baseline before CRRT was initiated. Therefore, losses from CRRT cannot be assumed to be the main reason for altered nutrient balance in severe AKI.

It is well known that Se, Zn, Fe and vitamin C homeostasis is altered in critical illness with redistribution from the blood to the tissues^22^. Vitamin D concentrations are also frequently low during critical illness, but less is known about the metabolism of vitamin D and other fat-soluble vitamins in AKI. Druml and colleagues measured plasma concentrations of vitamin A, E, D and K in AKI patients treated with haemodialysis and reported profound deficiencies with the exception of vitamin K.^23^ More research is ongoing^24^.

Besides their role as building blocks for proteins and hormones, amino acids are key regulators of metabolic pathways necessary for cell signaling, cell homeostasis, growth and immunity. Both, critical illness and AKI are pro-inflammatory conditions associated with an altered amino acid profile and increased protein catabolism^14,25,26^. Our study confirms that the concentrations of essential amino acids like arginine, histidine, lysine and tryptophan and glutamine were low in the majority of patients with severe AKI. We did not explore the effect on outcomes but previous studies in critically ill patients demonstrated an association between low glutamine concentrations and mortality^27^.

Carnitine, a 161 kDa amino acid derivate, has a key role in the transport of fatty acids from the mitochondrial intermembrane space into the mitochondrial matrix in skeletal and cardiac myocytes. In critically ill patients, carnitine deficiency has been associated with prolonged hospital stay^28,29^. Our study showed significantly lower carnitine concentrations in CRRT patients at 24 hours but not beyond.

Micronutrient deficiency has been associated with poor outcomes during critical illness ^30^. *In vitro* and animal studies suggest important roles for various nutrients. Recently, several investigators explored the role of vitamin c and thiamine supplementation during critical illness with particular emphasis on patients with sepsis^31–35^. However, the role of nutrient supplementation in patients with severe AKI has not been studied. With serial data of 33 different nutrients measured over a six-day period in 55 patients receiving modern critical care, this is the largest study to date investigating the nutrient profile in critically ill patients with AKI. Our results reject the commonly held view that losses during RRT are the main reason for altered micronutrient profile in patients with AKI.

It is important to acknowledge some potential limitations. First, in an intention-to-treat analysis, we compared patients who received CRRT on day of enrolment with non-CRRT patients. However, some patients recovered renal function during the study period, and similarly, a proportion of patients in the control group required CRRT later. While these confounding factors were unavoidable, we explored the influence of cross-over by comparing three patient groups based on treatment received. Second, we did not find significant differences between CRRT and non-CRRT patients but note that the mean and median values of most nutrients were lower in the CRRT cohort. This was an exploratory study and was not powered to be definitive. Adjusting for multiple testing will almost certainly render no differences statistically significant but this might be a type 2 error. Importantly, the absence of statistical significance should not be viewed as an indicator of lack of clinical significance. Third, we found low plasma concentrations of several different nutrients but are unable to comment on the underlying cause. Whilst we only included patients who were fully established on enteral nutrition, underfeeding may still have contributed to low nutrient concentrations in plasma. Fourth, we measured amino acid concentrations but did not measure any related proteins, peptides or hormones and cannot comment on the clinical impact of amino acid deficiency. Fifth, we did not control for underlying illnesses and acknowledge that plasma micronutrient concentrations may have been affected by systemic inflammation^21^. However, there were no significant differences in CRP, WBC and serum albumin between the CRRT and non-CRRT groups at 24 hours and at 6 days. Sixth, we measured vitamins B_1,_ B_6,_ B_12_, C and D and note that previous studies also reported deficiencies of vitamin A, B_3_, E and K^22,36^. Seventh, vitamin concentrations were evaluated on the basis of abundance in plasma or whole blood only. We did not assess vitamin utilization. Eighth, patients were enrolled in 2013/2014 but the laboratory and statistical analysis was delayed until sufficient funding was available for the detailed analyses. Nutrient concentrations were not affected by storage. We also believe that clinical practice and management of CRRT has not changed significantly since then and that our results remain highly relevant for current practice. Ninth, we analysed patients with severe AKI with and without CRRT but did not include a cohort of patients without AKI. When we conceptualised the study and drafted the study protocol, it was (and still is) common belief that CRRT is responsible for significant nutrient losses and nutrient deficiencies. We hypothesized that patients treated with CRRT had significantly lower plasma concentrations of water soluble nutrients than patients with similar degrees of AKI not treated with CRRT. To test this hypothesis, we compared two cohorts of patients who were similar apart from the intervention of interest. At that time, we had not expected that patients with AKI not treated with CRRT also had derangements of serum nutrient concentrations to such an extent as seen in our study. Finally, we are unable to make recommendations for changes in nutritional support for patients with AKI based on our observational data but feel that intervention studies are urgently required.

In conclusion, in critically ill patients with severe AKI, serum concentrations of important nutrients are commonly below the reference range, independent of treatment with CRRT. All amino acids, trace elements and water soluble vitamins were removed during CRRT but there were no statistically significant differences in plasma nutrient concentrations between the CRRT and non-CRRT group during a six-day follow-up period, except for glutamic acid. Future research should explore the role of nutrient supplementation in high risk patients with AKI.

## Methods

### Setting

This prospective observational study was conducted in multidisciplinary Intensive Care Units (ICUs) of two tertiary care hospitals in London, UK.

### Patient population

Between April 1, 2013, through April 1, 2014, we recruited consecutive critically ill adult patients (≥18 years) with AKI stage 2 or 3 as defined by the Kidney Disease Improving Global Outcome classification^21^. Accordingly, AKI stage 2 was defined by 2.0–2.9 fold rise of serum creatinine from baseline and/or urine output <0.5 ml/kg/hr for ≥12 hours; AKI stage 3 was defined by either a ≥3-fold rise of serum creatinine from baseline, a serum creatinine rise to ≥353.6 µmol/L (4.0 mg/dL), initiation of renal replacement therapy, anuria for ≥12 hours or a fall in urine output to <0.3 ml/kg/hr for ≥24 hours^21^. Exclusion criteria were: AKI stage 2/3 for more than 36 hours, pre-existing dialysis dependent renal failure, receiving supplementation with intravenous multivitamins or trace elements, need for total parenteral nutrition (TPN), or expected life expectancy <48 hours. We also excluded patients in whom serial blood sampling was not desirable (ie. Jehovah’s witness, patients with haemoglobin <70 g/L). Patients who needed RRT were treated with continuous veno-venous haemofiltration or continuous veno-venous haemodialysis using citrate or heparin anticoagulation.

All patients who were enrolled were established on full enteral feed receiving Nutrison Multifibre (1000 kcal/L; 123 g carbohydrate, 39 g fat and 40 g protein per litre; Nutricia Ldt, Trowbridge, UK) as per departmental protocol. Patients were allowed additional oral food intake if appropriate. Following routine individualized nutrition assessment by the departmental dietetic team, some patients were changed to Nutrison Protein Plus Multifibre (1280 kcal/L; 141 g carbohydrate, 49 g fat and 63 g protein per litre; Nutricia Ltd, Trowbridge UK), Nepro (2000 kcal/L; 206 g carbohydrate, 96 g fat and 70 g protein per litre) or Nepro HP (1800 kcal/l; 147 g carbohydrate, 97.7 g fat and 81 g protein per litre; Abbott Nutrition, Berkshire, UK) during the course of the study. All enteral feeds provided similar amounts of amino acids, vitamins and trace elements per 100 kcal. Patients who had to be commenced on TPN for clinical reasons during the 6-day follow-up period after enrolment were excluded from the primary analysis.

### Collection of samples

Blood samples were collected at baseline and 22–26 h, 46–50 h, 94–98 h and 142–146 h after enrolment for measurement of plasma concentrations of Se, Zn, Cu, iron (Fe), vitamins B_12_, C and D, folate, carnitine and 22 different amino acids (leucine, isoleucine, valine, glycine, alanine, methionine, arginine, citrulline, ornithine, proline, glutamic acid, glutamine, lysine, tryptophan, phenylalanine, tyrosine, hydroxyproline, threonine, serine, aspartic acid, taurine, histidine). Vitamin B_1_ and B_6_ concentrations were measured in whole blood.

In CRRT patients, effluent samples were collected at the same time points for measurement of the same nutrients. All samples were processed and stored in dedicated research freezers at −80 °C until batch analysis at the end of the study.

### Collection of clinical data

We collected baseline demographics, admission diagnosis, Acute Physiology and Chronic Health Evaluation (APACHE) II and Sequential Organ Failure Assessment (SOFA) scores on admission to ICU, daily SOFA score, type and amount of nutrition, daily C-reactive protein (CRP), white cell count (WBC) and serum albumin concentration, and ICU, hospital and 90-day outcome. In CRRT patients, we calculated the total dose of RRT delivered per 24-hour period. The total daily amount of nutrients lost in the effluent was calculated by multiplying the nutrient concentration in the effluent with the total dose of RRT delivered in 24 hours.

### Laboratory analyses

Se, Cu and Zn were measured by inductively-coupled mass spectrometry (ICP-MS) using the Thermo XSeries II instrument (Thermo Fisher Scientific, Hemel Hempstead, UK). Iron was measured using the colorimetric ferrozine method on the ADVIA 2400 analyser (Siemens Diagnostics, Frimley, UK).

Concentrations of vitamin B_1_ and B_6_ were determined by high-performance liquid chromatography (HPLC) with fluorescence detection (Chromsystems, Gafelfing, Germany), whilst vitamin C was measured by HPLC with ultra-violet (UV) detection. Folate and vitamin B_12_ were measured by immunoassay (Abbott Architect), and vitamin D_3_ concentrations were determined by liquid chromatography–mass spectrometry (LC-MS/MS) consisting of an Agilent 1260 LC system and a 6460 MS. Amino acids were measured underivatised by stable isotope dilution LC-MS/MS using an Agilent 1260/CTC-PAL LC system, with a Supelco Astec Chirobiotic T column (Sigma-aldrich, Poole, UK), and an ABSciex API6500 Qtrap MS/MS (ABSciex, Warrington, UK). The reference values of all nutrients are shown in Supplementary Table 1.

### Statistics

#### Sample size calculations and statistical analysis

It was estimated that a pilot sample of 40 patients (20 CRRT and 20 non-CRRT patients) would provide sufficient precision with 95% confidence interval (CI) for the change in plasma nutrient concentrations between pre-CRRT and 24–144 hours later being estimated to approximately ±0.4 standard deviations (SDs) of the change. When possible, we presented the estimated differences between the two groups with 95% CI. If estimated differences could not be calculated due to small numbers, a p-value was given.

The primary outcome was the difference in plasma concentrations of essential nutrients between the CRRT and non-CRRT cohort at 24 hours after enrolment. For continuous data, differences in the baseline characteristics of the CRRT and non-CRRT group were tested using t-test, or Mann-Whitney U tests where data were non-normal and could not be transformed to normal. Categorical data were compared using Chi-square tests (χ²) or Fisher’s exact test. SPSS (version 21) was used for the data analysis.

Limited comparison of nutrients against reference ranges were made across a six-day period for all patient groups. Continuous measures were reported with mean, SD and difference and 95% CI for the difference between groups using the t method. When test assumptions were not justified, either Mann-Whitney U or Wilcoxon test were applied to provide an indicative p-value with median and interquartile range (IQR). Categorical data were presented with total number (%) unless specified and compared with Chi-square (χ²) test, or when test assumptions were not justified, Fisher’s exact test to give indicative p values. Treatment groups were compared using Analysis of Variance (ANOVA), Fisher’s exact or Kruskall Wallis test where appropriate. If the difference between groups was statistically significant, *post hoc* analysis was performed using the Fisher’s exact pairwise comparison, Mann-Whitney U, or the Tukey honest significant difference test.

We compared patients according to treatment at enrolment (CRRT versus no CRRT) at 24 hours and six days after enrolment. In addition, we differentiated between 3 groups based on the treatment with CRRT during the six-day study period: i) patients who received CRRT every day; ii) patients who did not receive any CRRT during the six day follow-up period; and iii) a mixed group of patients who had CRRT on some but no all days.

### Ethics approval and consent to participate

The study was approved by the national Research Ethics Committee (REC) (REC number 13/LO/0064) and the institutional Research & Development Department. It was conducted in accordance with the Declaration of Helsinki and consent for participation was obtained. If possible, patients were asked to provide written informed consent prior to enrolment. If patients did not have capacity to consent, the opinion of a personal consultee was sought in accordance with section 32 of the Mental Capacity Act 2005 (UK). A personal consultee was a person who was engaged in caring for the participant (not professionally or for payment) and was interested in his/her welfare, and was prepared to be consulted. The personal consultee was asked to give an opinion as to whether the patient would object to taking part in medical research. If the personal consultee decided that the patient would have no objection to participating in the trial, he/she was asked to sign the Personal Consultee Declaration Form. As soon as the patient regained capacity, they were fully informed about the study and invited to give consent to continue participation. If the patient refused consent, all samples collected for research purposes were discarded and the patient’s data were not included in the analysis. If the patient did not regain capacity and/or died before consent could be sought, the REC felt it appropriate that the patient was included and that the samples were analysed provided the personal consultee did not indicate that the patient would not want to continue in this research project.

### Clinical trial registration

The study was registered with ISRCTN (ISRCTN88354940) and ClinicalTrials.gov (Identifier: NCT02470520).

## Supplementary information

Supplementary files.
