# Supplementary material for: Micronutrients in critically ill patients with severe acute kidney injury – a prospective study
Source: Sci Rep. 2020 Jan 30;10:1505. doi: 10.1038/s41598-020-58115-2 (PMC6992767; doi:10.1038/s41598-020-58115-2)
Supplement: Supplementary file 1 — Supplementary files. [file 41598_2020_58115_MOESM1_ESM.doc]

**Micronutrients in critically ill patients with severe acute kidney injury – a prospective study**

Marlies Ostermann, Jennifer Summers, Katie Lei, David Card, Dominic J Harrington, Roy Sherwood, Charles Turner, Neil Dalton, Janet Peacock, Danielle E Bear

**Supplementary Table 1: Reference ranges of measured vitamins, trace elements and amino acids**

|  | **Reference range** |
| --- | --- |
| **Trace elements** | |
| Fe | 11 – 29 μmol/L |
| Se | 0.89 – 1.65 μmol/L |
| Cu | 12 – 25 μmol/L |
| Zn | 11 – 19 μmol/L |
| **Vitamins** | |
| Vitamin D_3_ | >50 nmol/L |
| Folate | 3.1 – 20.5 μg/L |
| Vitamin B_12_ | 187 – 883 ng/L |
| Vitamin B_1_ | 66.5 – 200 nmol/L |
| Vitamin B_6_ | 35.2 – 110 nmol/L |
| Vitamin C | 26.1 – 84.6 μmol/L |
| **Essential amino acids** | |
| Leucine | 50 – 264 μmol/L |
| Valine | 96 – 566 μmol/L |
| Isoleucine | 26 – 159 μmol/L |
| Methionine | 10 – 53 μmol/L |
| Arginine | 43 – 407 μmol/L |
| Lysine | 119 – 233 μmol/L |
| Tryptophan | 40 – 91 μmol/L |
| Phenylalanine | 34 – 110 μmol/L |
| Threonine | 38 – 239 μmol/L |
| Histidine | 60 – 109 μmol/L |
| **Conditionally essential amino acid** | |
| Glutamine | 307 – 768 μmol/L |
| **Non-essential amino acids** | |
| Glycine | 81 – 303 μmol/L |
| Alanine | 112 – 686 μmol/L |
| Proline | 45 – 452 μmol/L |
| Glutamic acid | 21 – 174 μmol/L |
| Citrulline | 8 – 57 μmol/L |
| Ornithine | 27 – 83 μmol/L |
| Tyrosine | 26 – 154 μmol/L |
| Hydroxyproline | 4 – 27 μmol/L |
| Serine | 51 – 231 μmol/L |
| Aspartic acid | 1 – 4 μmol/L |
| Taurine | 19 – 173 μmol/L |
|  |  |
| Carnitine | 25 – 70 μmol/L |

Abbreviations: Fe = iron; Se = selenium; Cu = copper; Zn = Zinc

**Supplementary Table 2: Concentrations of essential vitamins, trace elements and amino acids in effluent fluid at 24 hours after starting continuous renal replacement therapy**

| **CRRT group (n=31)** | **Mean concentration (SD) in effluent at 24 hours** |
| --- | --- |
| **Amino acids ^a^** |  |
| Leucine | 112.5 (48.3) |
| Valine | 185.8 (68.0) |
| Isoleucine | 71.4 (27.7) |
| Glycine | 168.6 (58.4) |
| Alanine | 280.0 (104.8) |
| Methionine | 28.4 (11.3) |
| Arginine | 59.1 (22.7) |
| Citrulline | 19.0 (7.1) |
| Ornithine | 55.2 (24.0) |
| Proline | 109.0 (54.5) |
| Glutamic acid | 33.0 (3.5) |
| Glutamine | 409.8 (24.5) |
| Lysine | 160.1 (17.3) |
| Tryptophan | 14.7 (1.3) |
| Phenylalanine | 92.3 (9.3) |
| Tyrosine | 70.4 (6.2) |
| Hydroxyproline | 40.4 (5.9) |
| Threonine | 89.7 (7.6) |
| Serine | 74.2 (4.0) |
| Aspartic acid | 5.6 (1.3) |
| Taurine | 23.1 (25.5) |
| Histidine | 62.7 (27.0) |
| Carnitine | 38.6 (3.9) |
| **Trace elements** |  |
| Fe [μmol/L] | 0.1 (0.1) |
| Se [μmol/L] | 0.01 (0.01) |
| Cu [μmol/L] | 0.1 (0.02) |
| Zn [μmol/L] | 0.1 (0.2) |
| **Vitamins** |  |
| Vitamin D_2_ [nmol/L] | 0 (0) |
| Vitamin D_3_ [nmol/L] | 0 (0) |
| Folate [μg/L] | 3.1 (3.3) |
| Vitamin B_12_ [ng/L] | 0 (0) |
| Vitamin B_1_ [nmol/L] | 0 (0) |
| Vitamin B_6_ [nmol/L] | 0 (0) |
| Vitamin C [μmol/L] | 17.9 (18.3) |

Abbreviations: CRRT = continuous renal replacement therapy; SD = standard deviation ^a^ in μmol/L

**Supplementary Table 3: Serial trace element and vitamin concentrations and proportion of patients with nutrient deficiency**

| **Element / Vitamin** | **Time period** | **Patient group ^a^** | **Number of patients** | **Mean (SD)** | **Range** | **Proportion of patients with nutrient results below reference**  **range** |
| --- | --- | --- | --- | --- | --- | --- |
| Iron (Fe)  Reference range: 11-29 μmol/L | D0 | CRRT | 31 | 10.6 (12.8) | 1.5 to 59.3 | 67.7% |
|  |  | Non-CRRT | 24 | 12.2 (15.1) | 0.6 to 53.7 | 62.5% |
|  | D1 | CRRT | 30 | 10.2 (11.8) | 2 to 61 | 73.3% |
|  |  | Non-CRRT | 22 | 12.0 (14.5) | 2 to 52 | 63.6% |
|  | D4 | CRRT | 27 | 12.6 (21.9) | 2.2 to 118.4 | 77.8% |
|  |  | Non-CRRT | 20 | 8.8 (6.6) | 2.5 to 29.9 | 70.0% |
|  | D6 | CRRT | 25 | 8.4 (6.6) | 2 to 36 | 76.0% |
|  |  | Non-CRRT | 17 | 9.3 (7.4) | 2 to 32 | 76.5% |
| Selenium (Se)  Reference range: 0.89-1.65 μmol/L | D0 | CRRT | 31 | 0.621 (0.3) | 0.3 to 1.3 | 83.9% |
|  |  | Non-CRRT | 24 | 0.8 (0.4) | 0.19 to 2.2 | 75.0% |
|  | D1 | CRRT | 30 | 0.6 (0.3) | 0 to 1 | 33.3% |
|  |  | Non-CRRT | 22 | 0.8 (0.4) | 0 to 2 | 27.3% |
|  | D4 | CRRT | 27 | 0.8 (0.3) | 0.35 to 1.55 | 81.5% |
|  |  | Non-CRRT | 20 | 0.8 (0.4) | 0.39 to 2.44 | 55.0% |
|  | D6 | CRRT | 25 | 0.8 (0.3) | 0 to 2 | 68.0% |
|  |  | Non-CRRT | 17 | 0.9 (0.4) | 0 to 2 | 58.8% |
| Copper (Cu)  Reference range: 12-25 μmol/L | D0 | CRRT | 31 | 15.9 (5.1) | 5.7 to 26 | 25.8% |
|  |  | Non-CRRT | 24 | 16.0 (5.0) | 2.8 to 23.7 | 25.0% |
|  | D1 | CRRT | 30 | 16.7 (5.4) | 7 to 26 | 23.3% |
|  |  | Non-CRRT | 22 | 17.2 (4.0) | 9 to 24 | 13.6% |
|  | D4 | CRRT | 27 | 17.5 (5.2) | 8.1 to 25.6 | 18.5% |
|  |  | Non-CRRT | 20 | 18.1 (5.4) | 6.8 to 26.9 | 15.0% |
|  | D6 | CRRT | 25 | 17.4 (5.9) | 8 to 32 | 24.0% |
|  |  | Non-CRRT | 17 | 17.7 (5.0) | 5 to 25 | 5.9% |
| Zinc (Zn)  Reference range: 11.0-19.0 μmol/L | D0 | CRRT | 31 | 5.3 (3.0) | 1.8 to 15.5 | 90.3% |
|  |  | Non-CRRT | 24 | 5.4 (2.4) | 0.7 to 9.7 | 100% |
|  | D1 | CRRT | 30 | 5.4 (2.5) | 2 to 13 | 96.7% |
|  |  | Non-CRRT | 22 | 5.8 (2.4) | 2 to 10 | 100% |
|  | D4 | CRRT | 27 | 7.9 (2.9) | 3.4 to 17.1 | 92.6% |
|  |  | Non-CRRT | 20 | 7.2 (1.9) | 3.9 to 11.3 | 90.0% |
|  | D6 | CRRT | 25 | 8.8 (3.3) | 4 to 22 | 92.0% |
|  |  | Non-CRRT | 17 | 8.3 (2.4) | 5 to 12 | 88.2% |
| Vitamin D_3_  Reference range:  >50 nmol/L  <30 nmol/L (deficient);  30-50 nmol/L (insufficient) | D0 | CRRT | 31 | 22.0 (17.4) | 7 to 76.3 | <30: 77.4%  30-50: 16.1% |
|  |  | Non-CRRT | 24 | 23.7 (19.3) | 8.6 to 98 | <30: 75.0%  30-50: 16.7% |
|  | D1 | CRRT | 30 | 19.6 (13.7) | 7 to 65 | <30: 83.3%  30-50: 13.3% |
|  |  | Non-CRRT | 23 | 22.3 (12.2) | 8 to 61 | <30: 82.6%  30-50: 12.9% |
|  | D4 | CRRT | 27 | 23.6 (16.1) | 7 to 69 | <30: 70.4%  30-50: 25.9% |
|  |  | Non-CRRT | 20 | 25.1 (14.6) | 7.1 to 71.8 | <30: 80.0%  30-50: 10.0% |
|  | D6 | CRRT | 24 | 25.8 (14.6) | 7 to 64 | <30: 70.8%  30-50: 16.7% |
|  |  | Non-CRRT | 17 | 20.6 (8.1) | 8 to 34 | <30: 88.2%  30-50: 11.8% |
| Folate  Reference range: 3.1 - 20.5 μg/L | D0 | CRRT | 31 | 6.8 (5.2) | 1.8 to 20 | 29.0% |
|  |  | Non-CRRT | 24 | 6.7 (4.6) | 1.7 to 20 | 29.2% |
|  | D1 | CRRT | 30 | 5.6 (4.6) | 1 to 20 | 20.0% |
|  |  | Non-CRRT | 23 | 6.5 (4.5) | 2 to 20 | 8.7% |
|  | D4 | CRRT | 27 | 6.8 (4.9) | 3 to 20 | 3.7% |
|  |  | Non-CRRT | 20 | 6.9 (4.4) | 1.7 to 20 | 10.0% |
|  | D6 | CRRT | 24 | 8.1 (4.9) | 3 to 20 | 4.2% |
|  |  | Non-CRRT | 17 | 6.9 (3.6) | 2 to 14 | 11.8% |
| Vitamin B_12_  Reference range: 187 to 883 ng/L | D0 | CRRT | 31 | 878.2 (664.2) | 203 to 2000 | 0 |
|  |  | Non-CRRT | 24 | 1011.5 (704.5) | 157 to 2000 | 8.3% |
|  | D1 | CRRT | 30 | 1002.8 (645.8) | 184 to 2,000 | 3.3% |
|  |  | Non-CRRT | 23 | 1017.4 (706.2) | 155 to 2000 | 8.7% |
|  | D4 | CRRT | 27 | 1063.6 (670.2) | 149 to 2000 | 3.7% |
|  |  | Non-CRRT | 20 | 1085.7 (680.2) | 148 to 2000 | 5.0% |
|  | D6 | CRRT | 24 | 1037.6 (567.8) | 162 to 2000 | 4.2% |
|  |  | Non-CRRT | 17 | 969.1 (601.0) | 196 to 2000 | 0 |
| Vitamin B_1_  Reference range: 66.5 – 200 nmol/L | D0 | CRRT | 30 | 169.4 (111.5) | 79 to 565.7 | 0 |
|  |  | Non-CRRT | 23 | 137.9 (90.3) | 58.8 to 523.1 | 4.2% |
|  | D1 | CRRT | 29 | 166.3 (104.2) | 60 to 480.1 | 3.2% |
|  |  | Non-CRRT | 23 | 144.2 (82.3) | 70.1 to 422.2 | 0 |
|  | D4 | CRRT | 27 | 167.5 (85.5) | 58.1 to 374.7 | 3.7% |
|  |  | Non-CRRT | 20 | 167.4 (68.8) | 94.3 to 167.4 | 0 |
|  | D6 | CRRT | 21 | 171.9 (83.3) | 82.2 to 409.9 | 0 |
|  |  | Non-CRRT | 16 | 170.1 (62.2) | 100.9 to 312.2 | 0 |
| Vitamin B_6_  Reference range: 35.2 – 110.1 nmol/L | D0 | CRRT | 28 | 133.2 (110.8) | 42.8 to 455.5 | 0 |
|  |  | Non-CRRT | 21 | 98.2 (93.1) | 30.4 to 472.2 | 8.3% |
|  | D1 | CRRT | 27 | 122.3 (98.3) | 34.1 to 480.1 | 3.2% |
|  |  | Non-CRRT | 21 | 96.1 (68.0) | 32.3 to 258.7 | 4.2% |
|  | D4 | CRRT | 27 | 138.0 (188.3) | 40.6 to 1006.3 | 0% |
|  |  | Non-CRRT | 20 | 105.5 (49.0) | 51 to 238.5 | 0% |
|  | D6 | CRRT | 21 | 114.2 (55.1) | 46.7 to 280.6 | 0% |
|  |  | Non-CRRT | 15 | 106.5 (73.5) | 53.2 to 360.9 | 0% |
| Vitamin C  Reference range: 26.1 - 84.6 μmol/L  ≤11 μmol/L deficient | D0 | CRRT | 30 | 30.6 (38.5) | 3 to 189 | ≤11 μmol/L: 60.0% |
|  |  | Non-CRRT | 23 | 19.5 (20.7) | 3 to 95 | ≤11 μmol/L: 78.3% |
|  | D1 | CRRT | 29 | 19.6 (29.6) | 3 to 145 | ≤11 μmol/L: 82.8% |
|  |  | Non-CRRT | 22 | 16.8 (10.7) | 3 to 46 | ≤11 μmol/L: 81.8% |
|  | D4 | CRRT | 26 | 16.2 (17.0) | 3 to 93 | ≤11 μmol/L: 92.3% |
|  |  | Non-CRRT | 20 | 17.5 (11.3) | 3 to 49 | ≤11 μmol/L: 85.0% |
|  | D6 | CRRT | 24 | 15.3 (10.5) | 3 to 44 | ≤11 μmol/L: 87.5% |
|  |  | Non-CRRT | 18 | 18.3 (11.5) | 3 to 48 | ≤11 μmol/L: 77.8% |

Abbreviations: CRRT = continuous renal replacement therapy;

The data are presented as the mean (SD) or number/total number (%) unless specified.

^a^ as per clinical team on day of enrolment

**Supplementary Table 4: Serial amino acid concentrations and proportion of patients with amino acid deficiency**

| **Amino acid** | **Day** | **Patient group ^a^** | **Number of patients** | **Mean (SD)** | **Range ^a^** | **Proportion of patients with amino acid results below reference range** |
| --- | --- | --- | --- | --- | --- | --- |
| Taurine  Reference range: 19-173 μmol/L | D0 | CRRT | 31 | 54.7 (34.3) | 0.6 to 141.8 | 9.7% |
|  |  | Non-CRRT | 24 | 49.9 (32.4) | 13.4 to 157.8 | 4.2% |
|  | D1 | CRRT | 29 | 33.5 (18.4) | 10 to 96 | 17.2% |
|  |  | Non-CRRT | 24 | 36.9 (30.3) | 6 to 149 | 25.0% |
|  | D4 | CRRT | 27 | 28.2 (19.6) | 0.1 to 96.9 | 33.3% |
|  |  | Non-CRRT | 20 | 28.5 (21.0) | 2.3 to 91.3 | 40.0% |
|  | D6 | CRRT | 25 | 27.2 (18.6) | 5.3 to 87.4 | 36.0% |
|  |  | Non-CRRT | 17 | 37.4 (45.2) | 7.9 to 200.9 | 41.2% |
| Threonine  Reference range: 38-239 μmol/L | D0 | CRRT | 31 | 78.5 (35.4) | 22.9 to 171.1 | 3.2% |
|  |  | Non-CRRT | 24 | 71.2 (33.2) | 34.9 to 169.6 | 8.3% |
|  | D1 | CRRT | 30 | 78.9 (40.3) | 20 to 190 | 13.3% |
|  |  | Non-CRRT | 24 | 81.4 (49.7) | 30 to 241 | 8.3% |
|  | D4 | CRRT | 27 | 106.0 (52.8) | 38.1 to 238.7 | 0 |
|  |  | Non-CRRT | 20 | 108.9 (43.0) | 55.7 to 224.7 | 0 |
|  | D6 | CRRT | 25 | 82.8 (40.2) | 41.7 to 231.4 | 0 |
|  |  | Non-CRRT | 17 | 103.7 (31.6) | 56.8 to 166.5 | 0 |
| Serine  Reference range: 51-231 μmol/L | D0 | CRRT | 31 | 68.0 (29.7) | 29.8 to 178.6 | 22.6% |
|  |  | Non-CRRT | 24 | 71.2 (33.2) | 34.9 to 169.6 | 25.0% |
|  | D1 | CRRT | 30 | 71.0 (30.2) | 19 to 167 | 23.3% |
|  |  | Non-CRRT | 24 | 73.1 (26.6) | 33 to 120 | 16.8% |
|  | D4 | CRRT | 27 | 82.7 (26.4) | 52.2 to 181.5 | 0 |
|  |  | Non-CRRT | 20 | 84.3 (33.0) | 51.1 to 187.1 | 0 |
|  | D6 | CRRT | 25 | 82.8 (40.2) | 41.7 to 231.4 | 16.0% |
|  |  | Non-CRRT | 17 | 82.7 (31.3) | 38.5 to 182.7 | 5.9% |
| Glutamic Acid  Reference range: 21-174 μmol/L | D0 | CRRT | 30 | 69.8 (28.4) | 25 to 153 | 0 |
|  |  | Non-CRRT | 22 | 80.8 (38.8) | 40 to 192 | 0 |
|  | D1 | CRRT | 28 | 58.4 (21.4) | 28 to 102 | 0 |
|  |  | Non-CRRT | 23 | 76.5 (38.1) | 24 to 205 | 0 |
|  | D4 | CRRT | 27 | 68.7 (25.9) | 34.4 to 140.6 | 0 |
|  |  | Non-CRRT | 20 | 90.2 (34.3) | 27.7 to 139.3 | 0 |
|  | D6 | CRRT | 25 | 388.8 (113.1) | 264.9 to 769.4 | 0 |
|  |  | Non-CRRT | 16 | 98.2 (44.0) | 47 to 182 | 0 |
| Glutamine  Reference range: 307-768 μmol/L | D0 | CRRT | 31 | 450.3 (174.4) | 166.2 to 1032.3 | 16.1% |
|  |  | Non-CRRT | 24 | 405.6 (194.1) | 184.4 to 880.9 | 37.5% |
|  | D1 | CRRT | 30 | 385.3 (139.0) | 177 to 851 | 26.7% |
|  |  | Non-CRRT | 24 | 405.2 (148.8) | 224 to 878 | 25.0% |
|  | D4 | CRRT | 27 | 423.3 (111.6) | 221.4 to 673.5 | 3.7% |
|  |  | Non-CRRT | 20 | 405.4 (110.7) | 244.6 to 645.9 | 20.0% |
|  | D6 | CRRT | 25 | 388.8 (113.1) | 264.9 to 769.4 | 20.0% |
|  |  | Non-CRRT | 17 | 394.8 (90.1) | 244.9 to 565.1 | 23.5% |
| Proline  Reference range: 45-452 μmol/L | D0 | CRRT | 31 | 157.8 (73.5) | 33.8 to 320.3 | 3.2% |
|  |  | Non-CRRT | 24 | 170.5 (100.8) | 54.8 to 450.7 | 0 |
|  | D1 | CRRT | 30 | 128.6 (69.7) | 26 to 335 | 3.3% |
|  |  | Non-CRRT | 24 | 172.9 (101.5) | 77 to 468 | 0 |
|  | D4 | CRRT | 27 | 173.5 (71.7) | 85.4 to 438.5 | 0 |
|  |  | Non-CRRT | 20 | 175.8 (70.1) | 85.4 to 321.3 | 0 |
|  | D6 | CRRT | 25 | 144.0 (67.2) | 46.6 to 298.7 | 0 |
|  |  | Non-CRRT | 17 | 170.2 (66.4) | 86.9 to 330.2 | 0 |
| Glycine  Reference range: 81-303 μmol/L | D0 | CRRT | 31 | 212.0 (150.1) | 77 to 929 | 2% |
|  |  | Non-CRRT | 24 | 188.8 (127.9) | 57.2 to 651.5 | 2% |
|  | D1 | CRRT | 30 | 169.7 (72.5) | 74 to 428 | 2% |
|  |  | Non-CRRT | 24 | 190.0 (127.6) | 79 to 705 | 1% |
|  | D4 | CRRT | 27 | 205.0 (108.4) | 73.2 to 606.7 | 1% |
|  |  | Non-CRRT | 20 | 210.0 (100.5) | 80.5 to 407.5 | 1% |
|  | D6 | CRRT | 25 | 183.0 (75.3) | 92.6 to 384.2 | 0 |
|  |  | Non-CRRT | 17 | 189.7 (71.2) | 91.6 to 334.8 | 0 |
| Alanine  Reference range: 112-686 μmol/L | D0 | CRRT | 31 | 334.0 (171.7) | 77 to 793 | 2% |
|  |  | Non-CRRT | 24 | 262.3 (140.9) | 109.4 to 623.8 | 1% |
|  | D1 | CRRT | 30 | 268.4 (132.2) | 91 to 705 | 2% |
|  |  | Non-CRRT | 24 | 289.9 (278.6) | 104 to 1539 | 1% |
|  | D4 | CRRT | 27 | 270.5 (97.2) | 149.7 to 578.7 | 0 |
|  |  | Non-CRRT | 20 | 257.6 (85.2) | 158.1 to 549.4 | 0 |
|  | D6 | CRRT | 25 | 236.1 (89.1) | 94.2 to 460.6 | 4.0% |
|  |  | Non-CRRT | 17 | 255.4 (49.8) | 182.4 to 331.8 | 0 |
| Citrulline  Reference range: 8-57 μmol/L | D0 | CRRT | 31 | 23.9 (12.4) | 2.7 to 61.9 | 6.5% |
|  |  | Non-CRRT | 24 | 25.0 (14.1) | 3.0 to 55.8 | 8.3% |
|  | D1 | CRRT | 29 | 19.4 (9.4) | 2 to 42 | 10.3% |
|  |  | Non-CRRT | 24 | 26.1 (14.5) | 6 to 56 | 8.3% |
|  | D4 | CRRT | 27 | 22.1 (7.8) | 10.9 to 41.9 | 0 |
|  |  | Non-CRRT | 20 | 27.1 (14.0) | 6.1 to 62.0 | 5.0% |
|  | D6 | CRRT | 25 | 22.2 (8.0) | 9.9 to 41.7 | 0 |
|  |  | Non-CRRT | 17 | 26.2 (10.0) | 3.7 to 47.0 | 5.9% |
| Valine  Reference range: 96-566 μmol/L | D0 | CRRT | 31 | 185.6 (64.2) | 87 to 382 | 3.2% |
|  |  | Non-CRRT | 24 | 211.6 (111.2) | 97.0 to 575.8 | 0 |
|  | D1 | CRRT | 30 | 173.3 (77.0) | 67 to 431 | 16.7% |
|  |  | Non-CRRT | 24 | 205.7 (73.0) | 83 to 350 | 4.2% |
|  | D4 | CRRT | 27 | 225.4 (63.8) | 63.7 to 229.0 | 3.7% |
|  |  | Non-CRRT | 20 | 218.7 (47.5) | 132.3 to 312.8 | 0 |
|  | D6 | CRRT | 25 | 197.1 (48.9) | 114.8 to 302.5 | 0 |
|  |  | Non-CRRT | 17 | 211.2 (76.9) | 94.9 to 411.2 | 5.9% |
| Methionine  Reference range: 10-53 μmol/L | D0 | CRRT | 31 | 24.2 (10.4) | 8.9 to 47.3 | 6.5% |
|  |  | Non-CRRT | 24 | 26.3 (15.3) | 10.4 to 73.6 | 0 |
|  | D1 | CRRT | 30 | 25.8 (12.3) | 7 to 56 | 10.0% |
|  |  | Non-CRRT | 24 | 28.7 (20.1) | 11 to 88 | 0 |
|  | D4 | CRRT | 27 | 32.9 (21.4) | 10.2 to 125.7 | 0 |
|  |  | Non-CRRT | 20 | 32.2 (22.8) | 15.2 to 97.0 | 0 |
|  | D6 | CRRT | 25 | 26.3 (13.4) | 8.1 to 61.1 | 4.0% |
|  |  | Non-CRRT | 17 | 26.5 (8.2) | 9.2 to 43.9 | 5.9% |
| Isoleucine  Reference range: 26-159 μmol/L | D0 | CRRT | 31 | 56.8 (24.6) | 21 to 129 | 6.5% |
|  |  | Non-CRRT | 24 | 64.6 (39.0) | 19.7 to 201.2 | 8.3% |
|  | D1 | CRRT | 30 | 64.4 (33.1) | 20 to 150 | 13.3% |
|  |  | Non-CRRT | 24 | 64.0 (29.6) | 18 to 135 | 12.5% |
|  | D4 | CRRT | 27 | 70.2 (20.9) | 31.0 to 119.6 | 0 |
|  |  | Non-CRRT | 20 | 66.9 (16.4) | 38.8 to 96.7 | 0 |
|  | D6 | CRRT | 25 | 61.8 (14.1) | 31.6 to 90.5 | 0 |
|  |  | Non-CRRT | 17 | 65.6 (25.8) | 39.2 to 116.9 | 0 |
| Leucine  Reference range: 50-264 μmol/L | D0 | CRRT | 31 | 110 (42.0) | 44 to 226 | 3.2% |
|  |  | Non-CRRT | 24 | 130.7 (83.3) | 41.4 to 433.5 | 4.2% |
|  | D1 | CRRT | 30 | 120.3 (60.3) | 42 to 276 | 6.7% |
|  |  | Non-CRRT | 24 | 123.5 (48.0) | 41.8 to 240.3 | 4.2% |
|  | D4 | CRRT | 27 | 130.6 (36.7) | 63.7 to 229.0 | 0 |
|  |  | Non-CRRT | 20 | 123.7 (34.0) | 67.3 to 188.0 | 0 |
|  | D6 | CRRT | 25 | 112.5 (28.1) | 70.7 to 162.1 | 0 |
|  |  | Non-CRRT | 17 | 124.7 (50.5) | 61.3 to 235.0 | 0 |
| Tyrosine  Reference range: 26-154 μmol/L | D0 | CRRT | 31 | 63.8 (24.3) | 24.1 (116.5) | 9.7% |
|  |  | Non-CRRT | 24 | 66.5 (37.3) | 21.0 to 208.8 | 4.2% |
|  | D1 | CRRT | 30 | 63.9 (27.8) | 21 to 112 | 10.0% |
|  |  | Non-CRRT | 24 | 24.4 (13.7) | 29 to 181 | 0 |
|  | D4 | CRRT | 27 | 77.4 (27.4) | 22.8 to 124.1 | 7.4% |
|  |  | Non-CRRT | 20 | 64.5 (23.9) | 29.3 to 117.8 | 0 |
|  | D6 | CRRT | 26 | 74.5 (27.7) | 31.4 to 146.3 | 0 |
|  |  | Non-CRRT | 17 | 64.0 (25.2) | 31.4 to 115.4 | 0 |
| Phenylalanine  Reference range: 34-110 μmol/L | D0 | CRRT | 31 | 98.0 (54.4) | 36.7 to 282.6 | 0 |
|  |  | Non-CRRT | 24 | 128.9 (126.7) | 43.9 to 621.8 | 0 |
|  | D1 | CRRT | 30 | 90.3 (46.6) | 31 to 259 | 3.3% |
|  |  | Non-CRRT | 24 | 133.8 (175.2) | 47 to 919 | 0 |
|  | D4 | CRRT | 27 | 101.2 (38.1) | 57.6 to 214.9 | 0 |
|  |  | Non-CRRT | 20 | 132.3 (194.6) | 55.7 to 952.9 | 0 |
|  | D6 | CRRT | 25 | 99.6 (36.6) | 54.3 to 172.6 | 0 |
|  |  | Non-CRRT | 17 | 94.3 (32.5) | 45.6 to 161.4 | 0 |
| Arginine  Reference range: 43-407 μmol/L | D0 | CRRT | 31 | 43.1 (16.7) | 18.6 to 93.2 | 58.1% |
|  |  | Non-CRRT | 24 | 44.5 (27.2) | 14.4 to 136.3 | 70.8% |
|  | D1 | CRRT | 30 | 47.8 (23.0) | 18 to 131 | 43.3% |
|  |  | Non-CRRT | 24 | 52.0 (24.6) | 22 to 142 | 37.5% |
|  | D4 | CRRT | 27 | 61.0 (24.4) | 25.6 to 61.0 | 22.2% |
|  |  | Non-CRRT | 20 | 62.4 (21.5) | 38.7 to 119.6 | 15.0% |
|  | D6 | CRRT | 25 | 54.0 (20.2) | 21.3 to 104.6 | 32.0% |
|  |  | Non-CRRT | 17 | 65.2 (19.7) | 24.2 to 65.2 | 11.8% |
| Ornithine  Reference range:  27-83 μmol/L | D0 | CRRT | 31 | 61.3 (23.7) | 19.2 to 121.5 | 3.2% |
|  |  | Non-CRRT | 24 | 59.6 (34.3) | 15.6 to 146.2 | 16.7% |
|  | D1 | CRRT | 30 | 59.0 (22.6) | 16 to 104 | 10.0% |
|  |  | Non-CRRT | 24 | 64.1 (28.0) | 24 to 105 | 8.3% |
|  | D4 | CRRT | 27 | 81.5 (37.4) | 23.1 to 179.6 | 3.7% |
|  |  | Non-CRRT | 20 | 77.9 (35.2) | 30.8 to 182.3 | 0.0% |
|  | D6 | CRRT | 25 | 73.6 (31.1) | 28.9 to 150.6 | 0.0% |
|  |  | Non-CRRT | 17 | 73.1 (25.6) | 15.1 to 120.2 | 5.9% |
| Lysine  Reference range:  119-233 μmol/L | D0 | CRRT | 31 | 139.5 (56.7) | 56.2 to 325.5 | 48.4% |
|  |  | Non-CRRT | 24 | 147.6 (95.3) | 63.4 to 443.7 | 45.8% |
|  | D1 | CRRT | 30 | 150.8 (84.2) | 70 to 506 | 33.3% |
|  |  | Non-CRRT | 24 | 162.2 (96.7) | 65 to 463 | 33.3% |
|  | D4 | CRRT | 27 | 178.3 (76.3) | 79.4 to 389.8 | 18.5% |
|  |  | Non-CRRT | 20 | 169.7 (45.4) | 88.2 to 255.3 | 15.0% |
|  | D6 | CRRT | 25 | 155.4 (60.6) | 65.7 to 299.1 | 32.0% |
|  |  | Non-CRRT | 17 | 170.4 (51.8) | 78.1 to 272.8 | 11.8% |
| Tryptophan  Reference range:  40-91 μmol/L | D0 | CRRT | 31 | 21.4 (11.4) | 3.1 to 48.7 | 96.8% |
|  |  | Non-CRRT | 24 | 24.3 (13.8) | 3.3 to 64.7 | 91.7% |
|  | D1 | CRRT | 30 | 22.2 (11.8) | 2 to 50 | 86.7% |
|  |  | Non-CRRT | 24 | 24.4 (13.7) | 7 to 71 | 91.7% |
|  | D4 | CRRT | 27 | 32.1 (9.8) | 14.3 to 47.4 | 63.0% |
|  |  | Non-CRRT | 20 | 27.5 (9.8) | 7.2 to 44.3 | 90.0% |
|  | D6 | CRRT | 25 | 31.8 (9.9) | 10.8 to 46.1 | 76.0% |
|  |  | Non-CRRT | 17 | 27.9 (10.7) | 10.6 to 48.1 | 88.2% |
| Hydroxyproline  Reference range:  4-27 μmol/L | D0 | CRRT | 31 | 12.3 (10.3) | 0.7 to 41.2 | 12.9% |
|  |  | Non-CRRT | 23 | 10.0 (11.4) | 1.2 to 44.4 | 34.8% |
|  | D1 | CRRT | 30 | 7.7 (5.6) | 1.0 to 22 | 26.7% |
|  |  | Non-CRRT | 24 | 8.7 (8.3) | 0.0 to 37 | 25.0% |
|  | D4 | CRRT | 27 | 7.7 (9.9) | 1.0 to 54 | 33.3% |
|  |  | Non-CRRT | 19 | 6.1 (5.2) | 0.0 to 20 | 31.6% |
|  | D6 | CRRT | 25 | 6.2 (8.0) | 0.8 to 41.2 | 52.0% |
|  |  | Non-CRRT | 17 | 4.7 (3.2) | 1.1 to 11.9 | 47.1% |
| Aspartic Acid  Reference range:  1-4 μmol/L | D0 | CRRT | 31 | 7.3 (3.3) | 1.6 to 14.7 | 0.0% |
|  |  | Non-CRRT | 24 | 8,4 (3.3) | 3.9 to 17.2 | 0.0% |
|  | D1 | CRRT | 30 | 8.1 (5.7) | 3 to 32 | 0.0% |
|  |  | Non-CRRT | 24 | 7.6 (2.9) | 4 to 13 | 0.0% |
|  | D4 | CRRT | 27 | 7.5 (3.8) | 1.9 to 21.8 | 0.0% |
|  |  | Non-CRRT | 20 | 8.9 (4.2) | 2.3 to 19.2 | 0.0% |
|  | D6 | CRRT | 25 | 8.2 (4.9) | 2.5 to 28.2 | 0.0% |
|  |  | Non-CRRT | 17 | 9.5 (4.6) | 5.0 to 23.6 | 0.0% |
| Histidine  Reference range:  60-109 μmol/L | D0 | CRRT | 31 | 78.6 (34.1) | 38.6 to 213.2 | 32.3% |
|  |  | Non-CRRT | 24 | 67.9 (25.7) | 31.5 to 121.2 | 54.2% |
|  | D1 | CRRT | 30 | 68.9 (34.1) | 38.6 to 213.2 | 40.0% |
|  |  | Non-CRRT | 24 | 70.5 (60.5) | 31.5 to 121.3 | 58.3% |
|  | D4 | CRRT | 27 | 68.3 (25.6) | 34.8 to 156.5 | 44.4% |
|  |  | Non-CRRT | 20 | 59.1 to 20.2) | 33.5 to 126.8 | 65.0% |
|  | D6 | CRRT | 25 | 62.3 (24.8) | 33.7 to 149.6 | 64.0% |
|  |  | Non-CRRT | 17 | 57.9 (12.5) | 42.3 to 85.0 | 64.7% |
| Carnitine  Reference range:  25-70 μmol/L | D0 | CRRT | 31 | 86.4 (32.9) | 22.3 to 160.1 | 6.5% |
|  |  | Non-CRRT | 24 | 73.7 to 33.9) | 26.9 to 146.5 | 0.0% |
|  | D1 | CRRT | 30 | 50.6 (19.6) | 9 to 91 | 10.0% |
|  |  | Non-CRRT | 24 | 75.4 (39.8) | 19 to 188 | 4.2% |
|  | D4 | CRRT | 27 | 51.3 (27.1) | 6.5 to 126.6 | 18.5% |
|  |  | Non-CRRT | 20 | 52.2 (24.1) | 5.2 to 100.3 | 10.0% |
|  | D6 | CRRT | 25 | 56.8 (29.6) | 8.1 to 127.2 | 16.0% |
|  |  | Non-CRRT | 17 | 49.8 (18.6) | 16.4 to 80.3 | 11.8% |

Abbreviations: CRRT = continuous renal replacement therapy

The data are presented as the mean (SD) or number/total number (%) unless specified.

a as per clinical team on day of enrolment
